# 分子印迹纳米酶在生物传感领域中的应用进展

**DOI:** 10.3724/SP.J.1123.2025.06023

**Published:** 2026-02-08

**Authors:** Xuan ZHANG, Shucheng LIU, Jianming PAN

**Affiliations:** 江苏大学化学化工学院，江苏 镇江 212013; School of Chemistry and Chemical Engineering，Jiangsu University，Zhenjiang 212013，China

**Keywords:** 分子印迹技术, 纳米酶, 高选择性, 生物传感, molecular imprinting technology （MIT）, nanozyme, high selectivity, biosensing

## Abstract

酶作为生物催化剂，其核心优势在于高效催化和特异性底物识别，这些特性使其在生物化学过程中发挥着不可替代的作用。近年来，仿生酶体系的构建取得了显著的进展，研究者通过整合小分子化合物、脱氧核糖核酸及纳米材料等多元组分，成功地开发出具有优异性能的人工仿酶体系。这类体系不仅展现出良好的催化性能，还具有活性可控、易于修饰、稳定性高和可重复使用等显著优势。然而，纳米酶作为仿生酶体系的重要组成部分，虽然具有突出的催化活性，但其底物识别能力仍存在明显不足。为解决这一问题，研究者开发了分子印迹纳米酶，通过将纳米材料的催化性能与分子印迹的特异性识别机制相结合，实现了对目标分子的精准识别和选择性催化。这种新型仿生催化体系不仅解决了传统纳米酶在底物识别能力方面的不足，还展现出广阔的应用前景，为酶工程领域的发展提供了新的思路和方法。本文首先概述了纳米酶的基本特性，随后阐述了分子印迹纳米酶的常规制备工艺，并深入探讨了分子印迹对纳米酶催化性能的影响。通过典型实例分析，重点介绍了分子印迹纳米酶在生物传感领域的最新研究进展。最后，探讨了该领域面临的挑战及未来发展方向，旨在为分子印迹与纳米酶在生物传感中的应用提供理论参考和实践指导。

## Abstract (en)

Enzymes， as biological catalysts， have garnered significant interest due to their exceptional efficiency and specificity. However， the fragility of natural enzymes under varying temperature and pH conditions significantly restricts their broader utilization. In the past few years， noteworthy advancements have been achieved in creating biomimetic enzyme systems. Scientists have effectively designed artificial enzyme-mimicking systems that exhibit outstanding performance through the integration of various components， including small molecule compounds， deoxyribonucleic acid， and nanomaterials. These systems not only exhibit remarkable catalytic efficiency but also offer considerable benefits， such as adjustable activity， simplicity in modification， and enhanced stability and reusability. Nanomachines， as a new type of enzyme analogues， specifically refer to nanomaterials with enzyme-like catalytic functions. They have played a significant role in the development of biomimetic enzyme systems. Since the first report in 2007 that iron oxide nanoparticles have peroxidase （POD） mimicking activity， hundreds of nanomaterials have been confirmed to have catalytic activities similar to those of natural enzymes such as POD and oxidase （OXD）. These novel enzyme analogues not only exhibit a wide range of enzyme-like activities and structural similarity to natural enzymes， but also possess unique nanomaterial characteristics， making their catalytic activities controllable and stable. As effective substitutes for natural enzymes， nanomachines have been widely applied in fields such as biosensing， medical treatment， and environmental remediation. While every cutting-edge technology presents certain limitations， nanozymes are not an exception. They encounter notable challenges， especially concerning substrate selectivity， which is essential for effective targeted catalysis and widespread applicability. To address the aforementioned imitation， researchers have been investigating effective approaches to improve the catalytic selectivity of nanozymes. Primarily， two methods are utilized to achieve selective bioanalysis based on nanozyme catalysis： the first method involves merging nanozymes with biological recognition factors （such as natural enzymes， antibodies， DNA strands， and aptamers）， while the second focuses on developing nanozymes that possess intrinsic catalytic specificity through techniques like structure-mimetic design， surface modifications， or molecular imprinting. Incorporating external biological recognition elements can undermine both the stability and cost-effectiveness of nanozymes. Additionally， the methods available for the effective conjugation of nanozymes with biological components are still in their infancy. The creation of structure-mimetic nanozymes tends to be intricate and requires meticulous regulation. In contrast， a straightforward and accessible method for generating substrate recognition sites on nanozymes is the application of molecular imprinting technology （MIT）. MIT replicates interactions between enzyme substrates or antibody-antigen pairs to fabricate a cavity that is precisely shaped and sized for a particular template molecule， thus facilitating accurate molecular recognition. Due to its exceptional specificity， stability， and reproducibility， MIT is widely utilized in various fields such as biosensing， medical diagnostics， pharmaceutical assessment， sample preparation， and fluorescent detection. Moreover， the inherent advantages of molecularly imprinted polymers （MIPs）， such as their economical nature， exceptional selectivity， remarkable thermochemical resilience， and the removal of the need for biologically derived techniques， have rendered molecular imprinting a feasible strategy for mimicking the roles of natural enzymes. Natural enzymes exhibit substrate specificity primarily due to the three-dimensional structure of their active sites. These active sites are meticulously shaped to ensure a perfect match with the spatial configuration of the intended substrate. Following this concept， molecular imprinting nanoenzymes cleverly integrate molecular imprinting techniques with the properties of nanoenzymes， allowing biomimetic catalysts to retain catalytic selectivity while also demonstrating remarkable substrate specificity. This paper first summarizes the fundamental characteristics of nanozymes， then elaborates on the conventional preparation processes for molecularly imprinted nanozymes， and thoroughly explores the impact of molecular imprinting on the catalytic performance of nanozymes. Through an analysis of typical cases， the latest research advancements in molecularly imprinted nanozymes biosensing field are introduced. Finally， this paper discusses the challenges encountered and future development directions in this area， aiming to provide theoretical references and practical guidance for the application of molecular imprinting and nanozymes in biosensing.

蛋白质与核糖核酸（RNA）作为生命体催化过程的核心要素，构成了生物酶的主要结构基础，其独特的催化效率和底物特异性在生物代谢过程中发挥着不可或缺的作用。然而，在工业规模化应用场景下，天然酶存在诸多技术瓶颈：高昂的生产成本、环境适应性不足以及回收利用困难等问题，严重限制了其大规模应用。针对这一现状，科研人员将研究重点转向了具备仿酶催化功能的纳米级催化系统。其中，具有类酶催化特性的纳米材料——纳米酶，因其融合了生物酶的高效催化优势与纳米材料的独特结构特征，表现出优异的稳定性、可调节的催化活性以及良好的环境适应性^［1-3］^，正逐渐成为天然酶的有效替代品，在工业、农业、生物医药等多个领域获得日益广泛的关注与应用。值得注意的是，虽然纳米酶在部分催化指标上已超越天然酶，但由于多功能活性中心的精确构建技术尚未成熟，其在底物识别精度方面仍有待提升。

为突破纳米酶催化选择性提升的技术瓶颈，研究者一直致力于创新性解决方案的开发。在生物分析领域，现有改善纳米酶选择性的方法主要分为两大类别^［4］^：（1）将生物识别元件（包括天然酶、抗体、核酸序列、适配体等）与纳米酶进行功能性整合^［5，6］^；（2）借助结构仿生设计、表面功能化或分子印迹技术赋予纳米酶催化特异性^［7］^。然而，引入外源性生物识别元件通常会导致纳米酶体系稳定性降低且制备成本增加，同时生物元件与纳米酶的高效偶联技术仍存在诸多挑战。对于结构仿生型纳米酶而言，其制备工艺复杂，对过程控制要求极为严格。相对而言，运用分子印迹技术在纳米酶表面创建底物识别位点的方法具有适用性广和操作简便等显著的优势。2017年，Liu研究团队^［8］^通过创新性实验构建了一种具有核壳结构的分子印迹纳米酶，其催化效率显著提升了100倍，这一重要发现为高选择性人工酶的研究提供了全新的思路。随后，科研人员对纳米酶与分子印迹技术的协同应用产生了极大的关注，致力于构建具备类天然酶底物识别与转化功能的人工酶系统。天然酶之所以具有底物专一性，主要归因于其活性位点的三维空间结构，这些活性中心经过精确设计，能够与目标底物的立体构型实现精准契合。基于这一原理，分子印迹纳米酶将分子印迹技术与纳米酶特性进行巧妙结合，使仿生催化剂在保持催化选择性的同时，也具备了优异的底物特异性。本文首先概述了纳米酶的基本特性，随后阐述了分子印迹纳米酶的常规制备工艺，并从选择性和催化活性方面探讨了分子印迹对纳米酶性能的优化机制。通过典型实例分析，重点阐述了分子印迹纳米酶在生物传感领域的最新研究成果。最后，对分子印迹纳米酶的未来研究趋势进行了展望和总结， 旨在为研究人员提供参考，促进生物传感技术的发展。

## 1 纳米酶的基本特性

蛋白质构成的天然酶因其优异的底物选择性和生物催化性能，在医药、农业、化工及食品工业等领域中占据关键的地位。然而，这类生物催化剂在强酸、强碱环境及高温条件下极易丧失活性，且易被蛋白酶分解，这些固有缺陷限制了其实际应用范围。因此，开发兼具高效催化特性与优异稳定性的合成酶体系，以弥补天然酶的不足，已成为当前研究的重要方向。纳米酶作为一类新型酶模拟物，特指具有类酶催化功能的纳米材料。自2007年首次报道四氧化三铁（Fe_3_O_4_）纳米颗粒具有过氧化物酶（POD）模拟活性以来，数百种纳米材料被证实具有模拟POD、氧化酶（OXD）等天然酶的催化活性。这类新型酶模拟物不仅展现出广泛的类酶活性与天然酶的结构相似性，还具备独特的纳米材料特性，使其催化活性具有可调控性与稳定性。作为天然酶的有效替代品，纳米酶已在生物传感、医疗治疗及环境修复等领域获得了广泛的应用^［9］^。

尽管纳米酶展现出与天然酶相近的催化特性，但其研发与推广仍面临诸多难题。首先，相较于天然酶，纳米酶的催化效率普遍偏低。在纳米酶的发展过程中，众多学者专注于其催化性能的优化，研究表明，纳米酶的活性与其尺寸、形貌、成分、表面功能化以及环境因素（包括光照、pH值、温度、离子浓度和分子种类等）密切相关。其次，纳米酶的作用机制尚未完全阐明，这阻碍了其构效关系的深入理解。再次，天然酶具有显著的底物特异性，而纳米酶在这方面仍有待提升。虽然将天然酶与纳米酶结合可在一定程度上缓解这一问题，但天然酶的引入会影响整个催化体系的稳定性和经济性。因此，引入特定识别机制（如分子印迹技术）是设计高特异性纳米酶的有效途径^［10，11］^。

## 2 分子印迹纳米酶

### 2.1 分子印迹纳米酶常规制备方法

分子印迹纳米酶的制备过程依赖于模板分子、功能单体及交联剂在纳米酶表面的协同聚合反应^［12］^。这3种组分构成了分子印迹技术的核心要素，其合理选择直接影响印迹纳米酶的制备效率与功能特性。在分子印迹过程中，作为识别靶点的模板分子需具备特定的结构特征：其官能团既不能阻碍聚合反应，又需与功能单体产生稳定的配位作用，并在聚合体系中保持化学惰性。功能单体的选择标准主要基于其与模板分子间的特异性相互作用力，这种作用可通过供体-受体配对或抗体-抗原识别等机制实现。其中，甲基丙烯酸甲酯凭借其独特的氢键供受特性，成为该领域普遍采用的功能单体。此外，交联剂的合理使用能够构建具有特定刚性的聚合物网络，其类型与浓度显著调控着分子印迹体系的选择性识别与结合性能^［13］^。

分子印迹纳米酶的制备流程主要包括以下步骤：初始阶段需预先合成纳米酶作为基础材料，随后将特定的“3种分子印迹元素”与纳米酶进行混合，并通过触发聚合反应在其外围构建分子识别层。聚合反应的引发方式主要包含自由基聚合、光诱导聚合及电化学聚合3种途径。在后续步骤中，采用特定溶剂（如醇类或乙酸）对模板分子进行洗脱处理，从而在纳米酶表面形成与模板分子在空间构型、功能基团分布等方面相匹配的识别空穴。这种结构特征使得所制备的分子印迹纳米酶能够实现对目标分子的特异性识别与结合。该制备工艺主要依赖于纳米酶与分子印迹之间的协同作用，这种相互作用可分为共价结合与非共价结合两种类型。其中，共价结合涉及电子共享形成的化学键，典型实例包括生物素-亲和素相互作用、酰胺键合、叠氮化物反应以及金硫键等；而非共价结合则包括范德华力、静电引力、*π-π*堆积效应、氢键作用及疏水作用等不涉及电子转移的相互作用。鉴于其广泛适用性，非共价相互作用在分子印迹纳米酶的构建中得到了普遍应用。目前，该技术主要采用表面印迹和表位印迹两种策略。对于具有简单分子结构或线性构型的小分子蛋白，通常采用表面印迹法，将模板分子完全包埋于分子印迹层中。相较于传统印迹方法，表面印迹技术具有形成较薄印迹层和便于模板分子去除的优势^［14］^。鉴于可变蛋白质或微生物组织通常呈现复杂的结构特征和多样化的官能团分布，传统的表面印迹技术在应用上受到明显限制。这种限制主要源于纳米酶表面生成的厚重印迹层对催化活性的显著抑制作用。针对这一问题，研究者提出了表位印迹法作为制备分子印迹纳米酶的有效替代策略。该技术通过将模板分子的特定片段嵌入分子印迹聚合物中，同时保持大部分结构暴露于印迹层之外。这种印记方法不仅有助于生物大分子的选择性去除和特异性识别，更在模板片段的选择上提出了严格要求：所选片段需具备足够的独特性以防止交叉反应，同时应包含适宜的功能基团以确保与功能单体的有效相互作用^［15］^。从空间策略角度来看，各类分子印迹技术各具优劣，其选择应主要基于模板分子的尺寸特征与空间构型进行考量。

### 2.2 分子印迹对纳米酶催化性能影响

相较于天然酶，纳米酶普遍存在特异性不足的显著缺陷。将分子印迹聚合物与纳米酶复合，能够实现性能的协同增强：前者主要负责分子识别功能，后者则贡献催化放大效应。这种优势互补机制为微型化检测装置及即时诊断平台的开发奠定了技术基础。研究发现，通过优化底物界面特性或增强局部分析物富集度，分子印迹技术可有效调控纳米酶的催化性能，进而实现检测系统选择性与灵敏度的双重提升。

这种协同机制源于目标物、分子印迹聚合物与纳米酶之间的局部相互作用。当目标物与分子印迹聚合物结合时，它会在靠近纳米酶催化位点的区域受到空间限制，从而增加局部反应物浓度并加快反应速率。这种由距离驱动的增强显著提高了传感器的性能，如Chen等^［16］^将四氧化三锰纳米酶与分子印迹聚合物结合用于四环素（TC）检测，线性范围为0.01~20 μmol/L，检出限为5 nmol/L。Zhang等^［17］^采用Fe_3_O_4_纳米酶模拟POD活性进行探索研究，通过沉淀聚合法将丙烯酰胺、*N*-异丙基丙烯酰胺（NIPAAm）及交联剂*N，N*′-亚甲基双丙烯酰胺（MBAAm）等单体共同包覆于Fe_3_O_4_纳米颗粒表面。为避免氧化反应，实验体系未引入过氧化氢（H_2_O_2_），而选用2，2′-联氮双（3-乙基苯并噻唑啉-6-磺酸）二铵盐（ABTS）作为模板分子完成印迹过程。随着Fe_3_O_4_表面印迹聚合物（MIP）凝胶层厚度的增加，印迹材料逐渐沉淀析出。通过后续洗涤步骤去除ABTS分子后，材料表面形成了特异性结合空腔，这种经印迹处理的Fe_3_O_4_纳米酶被命名为A-MIP，其中“A”表示模板分子ABTS。同时，研究者在未加入ABTS的条件下进行对照实验，获得非印迹聚合物（NIP）。基于ABTS的负电特性，实验还引入了阳离子单体*N*-［3-（二甲基氨基）丙基］甲基丙烯酰胺（DMPA），分别制备了印迹型纳米酶（A-MIPpos）和非印迹型纳米酶（NIPpos）。为验证催化效果，研究者对原始Fe_3_O_4_纳米酶、NIPpos和A-MIPpos进行了性能对比测试。实验过程中，除ABTS外，还采用3，3′，5，5′-四甲基联苯胺（TMB）作为另一种底物来测定其选择性。动力学实验数据表明，在ABTS氧化反应中，A-MIPpos凝胶的速率常数达到0.11 min^-1^，较Fe_3_O_4_纳米酶（0.025 min^-1^）显著提高了4.3倍，这充分证实了分子印迹技术对催化活性的增强效果。此外，进一步分析显示，未经印迹处理的NIPpos凝胶（0.036 min^-1^）活性仅略高于Fe_3_O_4_，由此可推算出印迹效应使催化活性增强3倍（0.11/0.036），而静电作用导致的活性增长约为1.4倍（0.036/0.025）。

Zare-Dorabei课题组^［18］^构建了一种基于铈/铁普鲁士蓝衍生分子印迹（MI）二氧化钛-铁-氧化铈纳米酶（（MI） TiO_2_-Fe-CeO_2_）阵列，该阵列对支链氨基酸（包括缬氨酸、亮氨酸及异亮氨酸）表现出显著的特异性识别能力（图1a），鉴于这些氨基酸与败血症的密切关联，该阵列在疾病标志物检测领域展现出重要的应用价值。为进一步评估纳米酶的催化特性，研究人员构建了Fe-CeO_2_/MITiO_2_与酶动力学稳态参数的相关性模型，并选用TMB作为反应底物进行米氏动力学分析。通过米氏方程*v*_0_=（*v*_max_［S］）/（*K*_m_+［S］），确定了反应初始速率（*v*_0_）、最大反应速率（*v*_max_）、底物浓度（［S］）及米氏常数（*K*_m_）。实验表明，采用缬氨酸、亮氨酸和异亮氨酸为模板的分子印迹二氧化钛（MI TiO_2_）包覆于Ce［Fe（CN）_6_］^-^衍生的Fe-CeO_2_（分别记作Fe-CeO_2_/ValITiO_2_、Fe-CeO_2_/LeuITiO_2_和Fe-CeO_2_/ILeITiO_2_）表面上，其*K*_m_值分别为0.31、0.43和0.64 mmol/L。研究结果表明，*K*_m_值越小，纳米酶与TMB底物的结合能力越强，进而表现出更优异的催化性能（图1b和1c）。

**图1:**
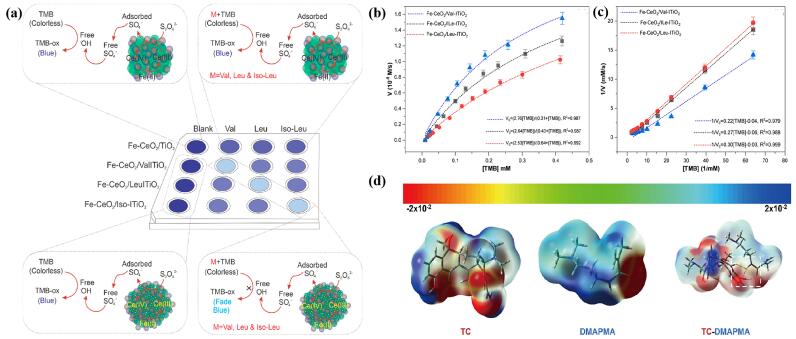
（a）缬氨酸、亮氨酸和异亮氨酸与所构建传感器在印迹型和非印迹型纳米酶中的作用机制^［18］^，不同氨基酸基分子印迹TiO_2_氧化TMB的（b）Michaelis-Menten曲线和（c）Lineweaver-Burk曲线^［18］^，（d） TC和DMAPMA分子静电图^［19］^

Liu等^［19］^开发了一种基于碱蚀技术的锰基普鲁士蓝类似物印迹材料，该材料具有优异的OXD模拟性能，能够对TC进行高特异性比色识别。在研究过程中，作者运用密度泛函理论对多种功能单体进行了全面筛选与优化。基于对称适应型微扰理论的密度泛函变体（density functional variant of symmetry-adapted perturbation theory，DFT-SAPT），分析了TC与各单体在1∶1（体积比）复合体系中的分子间相互作用能。研究表明，虽然TC与*N*-［3-（二甲基氨基）丙基］甲基丙烯酰胺（DMAPMA）之间存在显著的相互作用，但其主要驱动力源自二者正负静电位点的互补匹配，其中一阶静电贡献值达到-37.68 kcal/mol。分子静电势分布图（molecular electrostatic maps，MEP）进一步揭示，TC分子左下区域的羟基和羰基孤对电子呈现负电性特征，而DMAPMA分子右下端的胺基氢原子则表现出正电性（图1d）。位于左下角的TC分子中羟基氢表现出正电性，与之对应的DMAPMA分子在右下角羰基孤对电子处产生负电位，这些带正负电荷的区域构成了理想的氢键供体-受体对。TC-DMAPMA二聚体内部形成了3个氢键，其键长分别为1.70、2.05和2.18 Å，均分布在上述电荷区域。二阶响应极化，包括电场效应和电荷转移效应，对该二聚体产生了显著的感应（-13.85 kcal/mol）。此外，TC的多环构型与DMAPMA的长碳链结构共同导致了较大的色散作用（-22.45 kcal/mol）。基于计算结果可以推断，在众多功能单体中，DMAPMA对TC的识别能力最为突出。这种二聚体间强烈的结合作用为分子印迹技术的构建及其目标识别提供了有利条件。

为了进一步深入探究MIP对纳米酶催化性能的作用机制，Amine等^［20］^基于林韦伯-伯克方程（Lineweaver-Burk equation）对左旋多巴（L-DOPA）作为反应底物时的催化动力学进行了系统分析。实验数据表明，Fe_3_O_4_、Fe_3_O_4_-Lys-Cu及 Fe_3_O_4_-Lys-Cu@MIP（Lys为赖氨酸）在动力学参数上存在明显区别。动力学研究结果表明，Michaelis常数（*K*_m_）呈现Fe_3_O_4_-Lys-Cu@MIP<Fe_3_O_4_-Lys-Cu<Fe_3_O_4_递增趋势，而催化效率（*v*_max_/*K*_m_）则呈现相反的递减趋势（表1）。研究证实，通过氨基酸（如赖氨酸Lys）和铜离子（Cu）的修饰，可显著优化Fe_3_O_4_纳米酶的催化性能。其中，Lys分子中的氨基功能基团发挥了多重作用：增强底物结合能力、改善纳米颗粒分散性、抑制团聚现象并增大比表面积。与此同时，Cu离子凭借其过氧化物酶样特性，与Fe_3_O_4_纳米颗粒产生了协同催化效应。特别值得注意的是，Lys的氨基与L-DOPA分子之间可形成氢键及其他非共价相互作用，这种强化的分子间作用显著提升了Fe_3_O_4_-Lys与底物的结合效率。分子印迹技术的引入进一步优化了纳米酶的催化性能，这主要得益于MIP对L-DOPA分子特异性识别位点的构建。纳米酶与分子印迹聚合物之间的协同作用还增强了食品安全检测中的多组分分析能力。通过设计具有多个识别位点的MIP，并将其与具有广泛催化活性的纳米酶进行耦合，这些系统能够在单一平台上同时检测不同类别的污染物（例如抗生素、农药和毒素）^［21］^。纳米酶在催化和信号传导方面的双重作用与MIP的分子选择性相结合，使得能够开发出具有高度可靠且抗干扰的生物传感器。此外，分子印迹纳米酶混合系统通过印迹过程来调节纳米酶的反应活性，实现了可调的催化活性。纳米酶在MIP矩阵中的空间排列可以进行优化，以控制反应动力学，从而提高响应时间和信号与噪声比^［19］^。例如，具有分级孔结构的分子印迹纳米酶复合物能够促进目标分子的快速质量传递，进一步提高检测效率^［22］^，这一特性对于实时监测食品污染物至关重要。

**表1:** Fe_3_O_4_、Fe_3_O_4_-Lys-Cu以及Fe_3_O_4_-Lys-Cu@MIP的动力学参数^［20］^

Kinetic parameter	Fe_3_O_4_	Fe_3_O_4_-Lys-Cu	Fe_3_O_4_-Lys-Cu@MIP	With pre-concentration Fe_3_O_4_-Lys-Cu@MIP
*v*_max_/（mmol/min）	0.39	0.52	0.55	0.68
*K*_m_/（mmol/L）	1.50	1.60	1.25	0.06
*v*_max_/*K*_m_	0.26	0.325	0.44	11.33

在不同的研究中，纳米酶经过分子印迹处理后的选择性提高程度差异很大。一些报道表明分子印迹纳米酶对模板分子的催化活性是非模板分子的数十倍，而另一些则不到两倍。分子印迹纳米酶的催化选择性不仅取决于纳米酶的类型和印迹材料，还取决于合成过程中纳米酶与模板分子和功能单体的比例。这些变量与印迹层厚度和纳米酶表面形成的空腔数密切相关。到目前为止，分子印迹纳米酶与相应的天然酶之间缺乏全面的比较。虽然分子印迹技术可以提高纳米酶的催化选择性，但所获得的选择性性能与天然生物酶相差甚远。对于真实的复杂矩阵，其抗干扰能力能否达到应用水平仍是未知的。

### 2.3 分子印迹纳米酶在生物传感领域中的应用

近年来，纳米酶催化性能优化研究持续受到广泛关注，其中提升底物识别精准度成为重点研究方向之一。在这一背景下，分子印迹技术因其独特的模板识别机制而备受青睐。该技术通过构建特定模板结合位点，能够有效模拟天然酶与底物之间的分子识别作用机制。这种仿生策略使得纳米酶能够实现目标分子的特异性识别，从而显著提升其底物选择性和催化效率。研究表明，分子印迹技术的应用不仅优化了纳米酶的催化性能，还为其在生物传感、环境治理和医学诊断等领域的实际应用提供了新的可能性。

生物传感系统主要由分子识别模块和信号转换模块组成，其中识别模块通过特异性生物识别元件（如抗体或适配体）实现对目标物的精准捕获。检测过程的准确性和特异性主要取决于氢键作用、静电相互作用及疏水效应等分子间作用力。信号转换模块则用于将识别事件转化为可检测的物理信号，这一转换过程通常通过酶促反应诱导颜色或荧光变化来实现，并可通过催化循环机制放大信号，从而改善检测的灵敏度。相较于传统生物大分子，分子印迹纳米酶在分析检测应用中展现出显著优势，包括成本效益高、稳定性优异以及设计灵活性大等特点。目前，基于分子印迹纳米酶构建不同的传感技术已用于各种分析物检测，包括毒素^［23，24］^、抗生素^［16，25，26］^、农药残留^［27，28］^和生物活性物质^［29-34］^等（表2）。在本节我们根据不同的信号响应重点介绍了比色检测、荧光分析、电化学测量以及多模式检测等。

**表2:** 基于分子印迹纳米酶生物传感用于分析物检测

Material	Methods	Analyte	Linear ranges	LODs	Ref.
AuNP/Co_3_O_4_@Mg/Al cLDH	enzyme-linked immunosorbent assay	saxitoxin	0-1000 ng/mL	3.17 ng/mL	［^23^］
MIP/Mn-CeO_2_	electrochemical	deoxynivalenol	0.01-50 ng/mL	0.003 ng/mL	［^24^］
MIL-101（Fe）-NH_2_@MIP	ratiometric fluorescent， colorimetric	chloramphenicol	0.5-10 μmol/L， 10-100 μmol/L	0.84 μmol/L， 1.47 μmol/L	［^25^］
DSMIP@Mn_3_O_4_	colorimetric， electrochemical	tetracycline	0.5-150 μmol/L， 0.01-20 μmol/L	0.1 μmol/L， 5 nmol/L	［^16^］
Fe_3_O_4_@MIP	colorimetric	tetracycline	2-225 μmol/L	0.4 μmol/L	［^26^］
CeO_2_@PDA@AuNCs-MIPs	fluorescent	methyl paraoxon	0.45-125 nmol/L	0.15 nmol/L	［^27^］
MIP/Mn@NC	electrochemiluminescence，colorimetric	phoxim	0.05-5000 ng/mL， 5-1000 ng/mL	0.011 ng/mL， 1.27 ng/mL	［^28^］
MTi-MIP	colorimetric	glycoproteins	10-130 µg/mL	0.052 μg/mL	［^29^］
Fe-MOF@CMIP	fluorescence	triclocarban	10 pmol/L-3.5 nmol/L	8.5 pmol/L	［^30^］
CeO_2_HNPs@GOx-MIPs	cascade enzyme system	*β*-D-glucose	1.2 μmol/L-0.8 mmol/L	1.0 μmol/L	［^31^］
Fe_3_O_4_@Au-GOx-MIPs	electrochemical	*β*-D-glucose	10.0 μmol/L-5.0 mmol/L	5.0 μmol/L	［^32^］
MIP-aptamer-Fe_3_O_4_ NP	colorimetric	thrombin	108.1 pmol/L-2.7×10^-5^ mol/L	27.8 pmol/L	［^33^］
MIL-101（Co，Fe）	ratiometric fluorescence， colorimetric	vanillin	0.5-55 μmol/L， 1-50 μmol/L	104 nmol/L， 198 nmol/L	［^34^］

基于显色反应的比色检测构成了纳米酶传感体系的重要技术路径。通过纳米酶的催化功能，可激活TMB、ABTS等显色底物发生颜色变化，从而获取检测信号。在比色传感领域，分子印迹技术与纳米酶的结合显著提升了检测的特异性和灵敏度，使其在分析检测中获得普遍应用。例如，Zhang等^［29］^依据酶联免疫吸附试验（ELISA）的基本机制，构建了一种基于卫星构型的三明治比色检测系统，用于糖蛋白的可视化分析。该系统的核心组件是磁性二氧化钛分子印迹纳米颗粒（Fe_3_O_4_@TiO_2_@MIP），其作为“初级识别单元”，实现了目标分子的高效捕获与磁分离。此外，该研究团队还通过硼酸盐衍生物与聚丙烯酸的竞争性修饰，成功制备了Janus金纳米粒子基纳米酶（J-Au），该材料兼具靶标识别与催化增强双重功能。其中，J-Au模拟POD活性，充当“信号输出单元”，在糖蛋白的二次识别过程中发挥作用，并催化TMB氧化以生成可检测信号。以转铁蛋白为代表性糖蛋白进行验证时，该三明治组装体系展现出良好的可视化检测性能，其检出限为0.052 μg/mL，分析时间为40 min。相较于传统ELISA技术，该策略具有显著的成本优势与操作便捷性，无需依赖天然抗体或精密仪器即可完成检测任务。此外，Li团队^［35］^开发了一种基于分子印迹纳米酶的智能手机比色检测技术，该技术专用于红霉素的精准检测。通过硅烷偶联剂对纳米酶表面进行功能化修饰，结合分子印迹技术，成功实现了抗生素的特异性捕获。该平台在中性pH条件下表现出显著的选择性识别性能，为红霉素的快速检测提供了有效解决方案。该检测原理依赖于H_2_O_2_对TMB的催化氧化反应，在罗丹明B存在时，溶液颜色由蓝色转为紫色，这种显色变化可通过肉眼直接观察。该传感平台的线性检测范围为15~135 μmol/L，最低检出限为1.78 μmol/L，且对红霉素具有优异的选择性。在实际样品水和牛奶检测中，回收率为95.57%~103.20%。基于上述实验结果，该检测方法具有良好的实际应用价值，为便携式视觉检测技术的发展提供了创新性解决方案。

在多元复杂体系中实现葡萄糖的高灵敏度检测，对食品加工工艺优化与健康监测具有重要的指导价值^［36］^。当前基于天然酶的葡萄糖传感方法普遍存在成本高昂且稳定性不足的缺陷。Yu等^［37］^开发了一种基于金纳米粒子与聚多巴胺复合的分子印迹材料（Au@PDA-MIPs）。该研究以硼酸衍生物为功能单体，葡萄糖为模板分子，在Au@PDA纳米颗粒表面成功构建了分子印迹聚合物层。实验数据表明，该纳米酶的催化活性仅降低6.3%，但其选择性显著提升了230%以上。该传感器在10 μmol/L~1 mmol/L的葡萄糖浓度区间内展现出优异的检测性能，其检出限达到0.227 μmol/L（信噪比为3），有效地避免了其他单糖的干扰。此外，该传感器在回收率测试和长期稳定性评估中均表现出理想的结果。Liu等^［19］^专注于碱蚀锰基普鲁士蓝类似物（Mn-PBA）以增强OXD类活性，线性范围为0.2~200 μmol/L，检出限为0.07 μmol/L，同时具有出色的选择性、稳定性和在环境和食品样本中的可重复使用性（图2）。尽管所构建的技术体系能够实现对亚微摩尔至微摩尔级TC的定量检测，但其性能仍存在优化空间：首先，目标物的识别过程耗时较长，可考虑引入静电作用等分子间相互作用力以提升识别效率；其次，与荧光检测等技术相比，现有比色法的灵敏度相对不足，这限制了该方法在环境和食品样品中的实际应用，使其仅能实现微摩尔级的检测，而难以满足痕量分析的需求。因此，为了改善检测系统的整体性能，有必要引入具有更低检出限的分析技术。

**图2:**
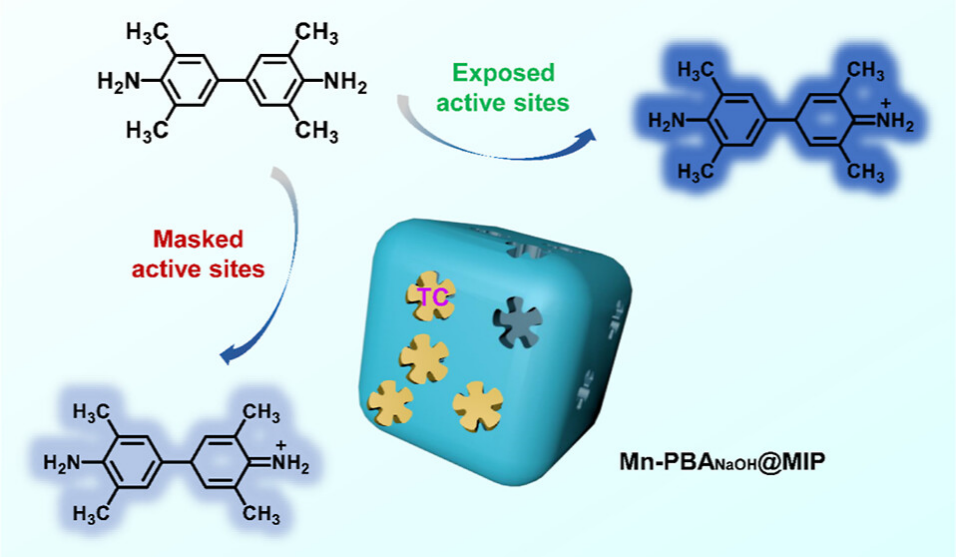
碱蚀刻的分子印迹型锰基普鲁士蓝类似物（Mn-PBA_NaoH_@MIP）用于TC检测的示意图^［19］^

与比色分析法相比，荧光检测被认为对目标传感具有更高的灵敏度。将分子印迹纳米酶与荧光测量相结合，可以实现荧光印迹传感器的高灵敏度和高选择性。例如，Wang等^［38］^将双功能铁基金属有机框架（NH_2_-MIL-101（Fe））与分子印迹技术相结合，成功构建了一种新型比率荧光探针，用于多巴胺（DA）的定量检测。该探针体系利用NH_2_-MIL-101（Fe）在452 nm处荧光发射特性，结合其优异的类POD活性，可催化邻苯二胺（OPD）转化为2，3-二氨基苯嗪（DAP），后者在556 nm处产生特征荧光信号。通过内滤效应（IFE）机制，这两个荧光信号实现了相互关联，从而构建出高灵敏度的复合检测系统。与传统的抗体识别方法相比，分子印迹技术的引入使DA分子探针的特异性和选择性能得到显著的改善。该荧光传感器在0.01~10 μmol/L的DA浓度范围内呈现良好的线性关系，其检出限为8.2 nmol/L。值得注意的是，在实际复杂基质样本的测试中，该传感器仍保持可靠的检测性能，充分证明了其在实际应用中的可行性与潜在价值。

目前，基于电化学的分子印迹纳米酶分析原理有两种：一种是将识别的目标在纳米酶的催化下分解产生电化学信号，另一种是利用一些电活性物质（TMB、ABTS等）作为自由标签，反映目标吸附在分子印迹纳米酶上引起的电化学界面变化。例如，Ma等^［39］^设计了一种三明治构型的电化学传感平台（图3），该平台将细菌印迹聚合物（BIP）与经万古霉素（Van）修饰的二氧化锰（MnO_2_）纳米酶（Van@BSA-MnO_2_）相结合，用于金黄色葡萄球菌（*S. aureus*）检测。借助电极表面原位合成技术，BIP能够精确复制目标细菌的表面特征，从而实现对目标菌株的特异性捕获。通过利用Van与细菌细胞壁之间的特异性结合作用，研究者使用1-（3-（二甲氨基）丙基）-3-乙基碳二亚胺盐酸盐（EDC）和*N*-羟基琥珀酰亚胺（NHS）作为交联剂，将Van与牛血清白蛋白（BSA）修饰的MnO_2_纳米酶进行共价连接，成功地制备了Van@BSA-MnO_2_信号探针。当该探针与目标细菌结合后，能够催化底物进行氧化还原反应，其产生的电信号强度与细菌数量之间表现出显著的线性相关性。在磷酸盐缓冲溶液的环境下，这一检测系统展现出优异的灵敏性，甚至能够实现单个细菌的精确检测。该检测系统无需对牛奶样本进行烦琐预处理即可稳定识别10 CFU/mL的金黄色葡萄球菌，体现了其优异的基质适应性。选择性实验表明，该系统能够准确识别目标菌，即使存在百倍浓度的同属干扰菌株也不影响检测结果。这一革兰氏阳性菌检测方案具有经济性和高效性双重特征。相较于传统纳米酶生物传感器，该方法不仅提升了检测灵敏度，简化了制备流程，更实现了成本的有效控制，这些特性使其在食品安全监管和环境中微生物检测方面具有重要的实践意义。

**图3:**
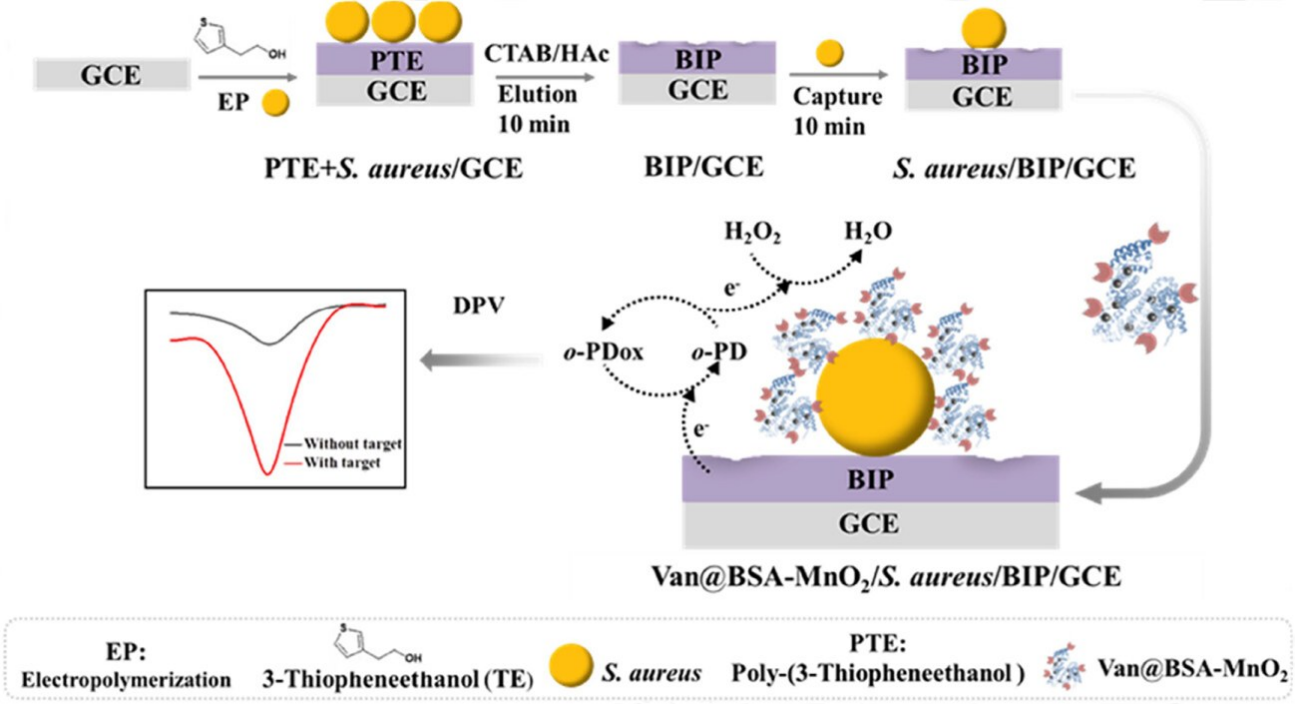
用于*S. aureus*检测的电化学传感器构建和工作原理示意图^［39］^

由上述可知，纳米酶催化产生的响应很容易转化为光学或电化学信号。近年来，多模式传感已成为分析化学和生物分析化学领域的研究热点。与单模测量相比，多模检测可以为同一反应过程提供一个以上的信号，这将从以下两个方面有利于检测：（1）可以根据现有条件选择合适的信号读取方式来实现检测需求，这对于资源有限的地区尤为重要；（2）不同的信号可以相互认证和校准，减少误差，提高检测精度。此外，还可以利用一些信号进行逻辑运算，提高检测灵敏度。如图4所示，Shen等^［40］^开发了一种基于MIPs和适配体的双重识别策略，成功设计出具有荧光-光电化学（FL-PEC）双信号响应特性的检测平台。在该系统中，荧光碳点（CDs）表面修饰的MIPs（CDs@MIPs）作为主要识别元件，用于捕获目标分子并抑制光诱导电子转移（PET）效应，从而触发荧光信号恢复。与此同时，适配体功能化脂质体（Apt@Lip-K_3_［Fe（CN）_6_］）作为辅助识别单元，其内部负载的六氰合铁酸钾（K_3_［Fe（CN）_6_］）可与CDs@MIPs/目标复合物发生特异性结合。脂质体破裂后释放的K_3_［Fe（CN）_6_］作为电子受体，能够显著提升CTAB@MAPbI_3_/ITO的光电流响应，由此产生第二个光电化学信号（PEC信号）的增强效应。以邻苯二甲酸二丁酯（DBP）为模型分析物，该双模式检测系统分别在0.1 nmol/L~10.0 μmol/L（FL）和1.0 pmol/L~0.1 μmol/L（PEC）范围内呈现良好的线性关系，检出限分别为71.30 pmol/L（FL）和0.648 pmol/L（PEC）（信噪比=3）。MIPs-适配体协同识别机制实现了快速可视化筛查（FL）与精确定量分析（PEC）的双重响应，显著提高了检测的灵敏度，同时有效降低了假阳性的发生。研究结果表明，这一系统在双酚A（BPA）等小分子酚类污染物的检测中表现突出，充分证明了其在食品与环境监测领域的广泛适用性和应用潜力。与此同时，Liu等^［41］^设计了一种基于铜基金属有机框架（NH_2_-CuBDC）纳米酶的双模式检测平台，成功实现了对有机磷农药的精确识别与定量分析。该团队基于该材料独特的类POD活性与荧光性质，构建了毒死蜱的比色-荧光双通道检测平台。实验结果表明，该检测体系具有显著的分析性能，其比色检出限为1.57 ng/mL，而荧光检出限为2.33 ng/mL（信噪比为3）。通过实际样品测试，证实了该方法在毒死蜱检测中具有良好的准确性与稳定性。该工作不仅拓展了纳米酶的应用领域，更为多模式传感技术的发展提供了理论支撑和实践依据。

**图4:**
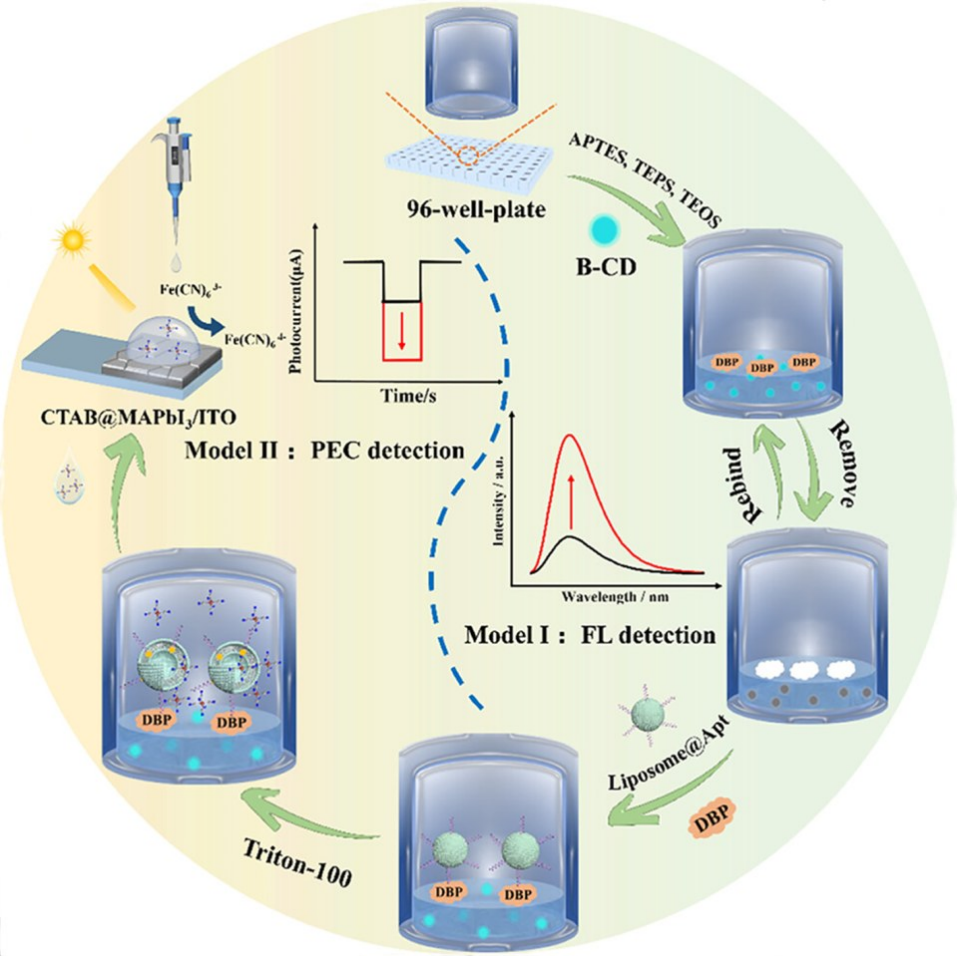
用于检测邻苯二甲酸二丁酯的荧光（模型Ⅰ）和光电化学（模型Ⅱ）传感平台的策略^［40］^

分子印迹纳米酶尽管在分析领域的应用取得了显著突破，然而其在实际应用中仍面临复杂基质中结构类似物干扰的挑战。为提升分子印迹聚合物的选择性，研究者们采用了多功能单体整合及后印迹修饰等前沿合成策略。这些技术不仅增强了分子印迹聚合物的识别能力，还改善了其稳定性和可重复利用性。值得注意的是，环境参数（包括温度变化、pH波动以及基质干扰）对传感器性能的影响仍需重点关注。虽然相较于生物抗体，分子印迹聚合物表现出更优异的稳定性，但在复杂环境基质中，其识别效能可能受到限制，进而影响检测的灵敏度和选择性。

## 3 结论

尽管纳米酶研究已取得了显著的进展，相较于天然酶亦展现出独特优势。然而，开发性能与天然酶相媲美的人工模拟酶仍存在很大困难。分子印迹技术的应用有效改善了纳米酶在催化过程中的选择性不足，同时实现了对其活性的精确调控，这极大地提升了其在分析化学领域的应用价值，但该领域仍存在诸多关键问题有待解决，未来研究可以从以下几个方面展开：（1）现有研究主要集中在分子印迹聚合物对纳米酶催化性能的调控机制，而纳米酶对分子印迹聚合物的作用机制尚未明确。研究表明，分子印迹纳米酶的识别特性，如吸附效率、结合动力学及选择性，可能与传统分子印迹技术存在显著差异。深入探讨这一相互作用机制将是未来研究的重点。（2）在分子印迹纳米酶体系中，催化特异性与活性的协同优化是亟待解决的技术难题。构建高性能的纳米酶核心与精确调控印迹层厚度以最小化活性抑制，是开发理想分子印迹纳米酶的关键。（3）现有分子印迹纳米酶的合成策略各具特点，需根据目标应用、材料属性及经济性等因素进行综合评估，以确定最优制备方案；强化分子印迹纳米酶的抗干扰能力，确保在多样化环境参数下获得稳定且精确的检测数据；同时开发集成多模态、智能化及微型化的传感系统，以增强其适用性与功能多样性。（4）推动分子印迹纳米酶产业化应用的关键在于开发简便、高效且可规模化的制备工艺。传统酶制剂与催化剂已形成完善的制备体系与市场应用模式，这为新型分子印迹纳米酶的发展提供了重要参考。（5）未来在模拟酶的设计和开发过程中，可结合计算机模拟和人工智能等新技术，实现信息挖掘和科学知识的自动收集，优化模拟酶的构建方案，预测模拟酶的催化活性和空间结构，并根据催化反应机理指导模拟酶的设计和合成。

## References

[1] Wei H， Gao L， Fan K， et al . Nano Today， 2021， 40： 101269

[2] Wu J， Wang X， Wang Q， et al . Chem Soc Rev， 2019， 48（4）： 1004 30534770 10.1039/c8cs00457a

[3] Wei H， Wang E . Chem Soc Rev， 2013， 42（14）： 6060 23740388 10.1039/c3cs35486e

[4] Li X， Zhu H， Liu P， et al . TrAC-Trends Anal Chem， 2021， 143： 116379

[5] Liu J， Niu X . Chemosensors， 2022， 10（10）： 386

[6] He X， Luo Q， Guo Z， et al . J Mater Chem B， 2022， 10（35）： 6716 35133373 10.1039/d1tb02325j

[7] Somerville S V， Li Q， Wordsworth J， et al . Adv Mater， 2024， 36（10）： 2211288 10.1002/adma.202211288 37017492

[8] Zhang Z， Zhang X， Liu B， et al . J A C S， 2017， 139（15）： 5412 28345903 10.1021/jacs.7b00601

[9] Huang Y， Ren J， Qu X . Chemical Reviews， 2019， 119（6）： 4357 30801188 10.1021/acs.chemrev.8b00672

[10] Karrat A， Amine A . Biosens Bioelectron， 2024， 250： 116053 38266615 10.1016/j.bios.2024.116053

[11] Guo Z， Luo Q， Liu Z . Chem Eur J， 2022， 28（61）： e202202052 35924666 10.1002/chem.202202052

[12] Bu Z， Huang L， Li S， et al . Anal Bioanal Chem， 2024， 416（27）： 5859 38308711 10.1007/s00216-024-05183-2

[13] Chen L， Wang X， Lu W， et al . Chem Soc Rev， 2016， 45（8）： 2137 26936282 10.1039/c6cs00061d

[14] Dong C， Shi H， Han Y， et al . Eur Polym J， 2021， 145： 110231

[15] Dietl S， Sobek H， Mizaikoff B . TrAC-Trends Anal Chem， 2021， 143： 116414

[16] Chen Y， Xia Y， Liu Y， et al . Biosens Bioelectron， 2022， 216： 114650 36049348 10.1016/j.bios.2022.114650

[17] Zhang Z， Li Y， Zhang X， et al . Nanoscale， 2019， 11（11）： 4854 30820498 10.1039/c8nr09816f

[18] Dashtian K， Afshar Gheshlaghi F， Zare-Dorabei R， et al . ACS Appl Bio Mater， 2024， 7（5）： 3346 10.1021/acsabm.4c00297 38695543

[19] Liu B， Zhu H， Liu J， et al . ACS Appl Mater Interfaces， 2023， 15（20）： 24736 37163688 10.1021/acsami.3c02207

[20] Karrat A， Amine A . Biosens Bioelectron， 2024， 266： 116723 39222569 10.1016/j.bios.2024.116723

[21] Chen Y， Tang K， Zhou Q， et al . Anal Chem， 2023， 95（49）： 18139 38013435 10.1021/acs.analchem.3c03571

[22] Wei Y， Xu R， Wang X， et al . Sens Actuators B-Chem， 2025， 423： 136800

[23] Cho C H， Kim J H， Padalkar N S， et al . Biosens Bioelectron， 2024， 255： 116269 38579624 10.1016/j.bios.2024.116269

[24] Nie D， Zhu X， Liu M， et al . J Hazard Mater， 2024， 477： 135366 39088943 10.1016/j.jhazmat.2024.135366

[25] He X-Y， Wang Y， Xue Q， et al . Food Chem： X， 2025， 26： 102322 40104617 10.1016/j.fochx.2025.102322 PMC11914186

[26] Liu B， Zhu H， Feng R， et al . Sens Actuators B-Chem， 2022， 370： 132451

[27] Zhang X， Hao N， Liu S， et al . Talanta， 2024， 277： 126434 38879946 10.1016/j.talanta.2024.126434

[28] Wang X， Zang X， Wang X， et al . Chem Eng J， 2024， 500： 156817

[29] Zhang Y-D， Ma C， Shi Y-P . Chem Eng J， 2023， 454： 140495

[30] Chen Y， Tang K， Wang X， et al . Sens Actuators B-Chem， 2023， 382： 133543

[31] Huang C， Cheng Y， Zhang Y， et al . Sens Actuators B-Chem， 2023， 379： 133222 10.1016/j.snb.2022.133223 PMC9771590 36573100

[32] Cheng Y， Chen T， Fu D， et al . Talanta， 2022， 242： 123279 35149425 10.1016/j.talanta.2022.123279

[33] Shen M， Wang Y， Kan X . J Mater Chem B， 2021， 9（20）： 4249 34008694 10.1039/d1tb00565k

[34] Zhang Y， Feng Y-S， Ren X-H， et al . Biosens Bioelectron， 2022， 196： 113718 34673481 10.1016/j.bios.2021.113718

[35] Li T， Xiao L， Ling H， et al . Food Chem， 2024， 449： 139291 38608609 10.1016/j.foodchem.2024.139291

[36] Zhou H， Qiu H， Zhang J， et al . Coordin Chem Rev， 2024， 500： 215523

[37] Yu J， Chen T， Wen X， et al . Biosens Bioelectron， 2024， 253： 116169 38442620 10.1016/j.bios.2024.116169

[38] Wang L， Wen L， Zheng S， et al . Sens Actuators B-Chem， 2022， 361： 131688

[39] Ma Y， Lin X， Xue B， et al . Anal Chem， 2024， 96（21）： 8641 38716697 10.1021/acs.analchem.4c00755

[40] Shen Y， Feng J， Wang Z， et al . Anal Chem， 2025， 97（25）： 13487 40546225 10.1021/acs.analchem.5c01915

[41] Liu S， Zhou J， Yuan X， et al . Food Chem， 2024， 432： 137272 37657347 10.1016/j.foodchem.2023.137272

